# Organoid technology in female reproductive biomedicine

**DOI:** 10.1186/s12958-020-00621-z

**Published:** 2020-06-18

**Authors:** Heidar Heidari-Khoei, Fereshteh Esfandiari, Mohammad Amin Hajari, Zeynab Ghorbaninejad, Abbas Piryaei, Hossein Baharvand

**Affiliations:** 1 Department of Stem Cells and Developmental Biology, Cell Science Research Center, Royan Institute for Stem Cell Biology and Technology, ACECR, P.O. Box: 16635-148, Tehran, 1665659911 Iran; 2 Urogenital Stem Cell Research Center, Shahid Beheshti University of Medical Sciences, Tehran, Iran; 3 Department of Biology and Anatomical Sciences, School of Medicine, Shahid Beheshti University of Medical Sciences, P.O. Box: 19395-4719, Tehran, Iran; 4 Department of Developmental Biology, University of Science and Culture, Tehran, Iran

**Keywords:** Organoids, Reproductive organs, Reproductive medicine, Organoid-on-a-chip

## Abstract

Recent developments in organoid technology are revolutionizing our knowledge about the biology, physiology, and function of various organs. Female reproductive biology and medicine also benefit from this technology. Organoids recapitulate features of different reproductive organs including the uterus, fallopian tubes, and ovaries, as well as trophoblasts. The genetic stability of organoids and long-lasting commitment to their tissue of origin during long-term culture makes them attractive substitutes for animal and in vitro models. Despite current limitations, organoids offer a promising platform to address fundamental questions regarding the reproductive system’s physiology and pathology. They provide a human source to harness stem cells for regenerative medicine, heal damaged epithelia in specific diseases, and study biological processes in healthy and pathological conditions. The combination of male and female reproductive organoids with other technologies, such as microfluidics technology, would enable scientists to create a multi-organoid-on-a-chip platform for the next step to human-on-a-chip platforms for clinical applications, drug discovery, and toxicology studies. The present review discusses recent advances in producing organoid models of reproductive organs and highlights their applications, as well as technical challenges and future directions.

## Background

The female reproductive system is of utmost importance to a woman’s quality of life; it produces sex hormones and oocytes, provides the site for fertilization, and supports the fetal development [1, 2]. Diseases and disorders of the female reproductive system are not adequately studied, especially in the areas of endometriosis, gynecological cancers, sexually transmitted diseases (STDs), and pregnancy disorders (including intrauterine growth restriction, miscarriage, and recurrent miscarriage), in addition to medications that have deleterious effects on the reproductive system [2–5]. Limited access to reproductive material, especially those for maternal-embryo interactions, and unavailable reliable experimental models are serious challenges for studies in this field [6].

Conventional in vitro systems that include two-dimensional (2D) and three-dimensional (3D) cell cultures have serious pitfalls that make them unsuitable for studying the female reproductive system. The 2D culture of immortalized cell lines or those derived from tumors in reproductive organs have led to numerous insights about the biology and physiology of the reproductive system; their advantages include reproducibility and ease of access. However, they lack many complex features of in vivo microenvironments, including a cell-cell/cell-extracellular matrix (ECM), the display of a relatively homogenous phenotype, and are not representative of in vivo cellular diversity. Because of genetic alterations in cell lines, they fail to recapitulate key features of native tissues as well as different active cell signaling pathways and function from those in the native cells [7]. Moreover, rapid loss of phenotype and tissue-related functions occur following 2D culture of primary cells [8]. Therefore, 2D culture systems do not adequately show the natural 3D environment of cells; in turn, they fail to mimic in vivo cellular functions and signaling pathways, and may provide misleading and non-predictive data for in vivo responses. The 3D cell aggregates exhibit improved function, but lack cellular polarity and the 3D organization that is present in vivo. Other 3D culture systems include spheroids that often lack the capacity for self-renewal and differentiation due to the absence of relevant progenitor or stem cells [9, 10]. The organ explant or organotypic slice cultures that recapitulate the complex 3D architecture, cellular heterogeneity, and function of the native organ are very useful for the study of development and physiology, but they are limited by their inability to proliferate, their short-term nature, and cell phenotype drift due to improper orientation in culture media [11, 12].

The recently developed organoid technology provides new in vitro models to serve as both tissue and organ proxies to bridge the gap between in vitro and in vivo [13]. Organoids are self-organizing structures that recapitulate a numerous of biological and pathological features of organs such as spatially restricted lineage commitment, specific functions of the organ, multiple organ-specific cell types, and cell-cell and cell-matrix interactions. They represent genetic stability and maintain commitment to their tissue of origin during the long-term culture that allows for access to an unlimited source of material to support research in this field. Moreover, they can serve as substitutes for animal and in vitro models and provide the opportunity for high throughput studies, personalized medicine, drug and toxicity testing, disease modeling, and present a promising way for autologous transplantation. Organoids recapitulate features of different reproductive organs. In this regard, the uterus, fallopian tubes, and ovaries as well as trophoblast organoids have been created in the laboratory. In this review, we will discuss recent advances in producing organoid models of reproductive organs and highlight their applications, in addition to discussing technical challenges and future directions.

## Main text

### Ovarian organoids

Ovarian cancer represents the fifth leading cause of cancer deaths in women in the US [14]. Access to reliable experimental models that address clinical challenges, such as early detection, tumor recurrence, and acquired chemotherapy resistance, is a high priority in ovarian cancer research. Recently, successful generation ovarian cancer organoids was reported by two separate groups [15, 16]. These organoids recapitulate histological and genomic features of the lesions from which they were derived and represent intra- and interpatient heterogeneity [15, 16]. Moreover, ovarian cancer organoids display somatic mutations and amplifications/deletions [15] and recapitulated the parent tumor’s marker expression and mutational landscape [16]. Moreover, the organoids showed tumor-specific sensitivity to chemotherapeutic drugs and therefore provide a reliable preclinical tools for drug screening and discovery [15, 16]. Moreover, xenografting these organoids can provide an in vivo platform for drug screening [15]. Maru et al. have previously reported that ovarian organoids recapitulated mutation profile and intra-tumor heterogeneity [17].

Developing organoids from other ovarian disorders such as premature ovarian failure and polycystic ovary would provide a platform to study the mechanisms that underlie these diseases and enable the development of new drugs and treatments.

### Fallopian tube organoids

The fallopian tube (oviduct or uterine tube) is a central organ of the female reproductive system that plays an essential role in oocyte maturation and selection, gamete and embryo transportation, sperm reservoir, control of polyspermy, fertilization and early embryonic development [18]. It is believed that the fallopian tube is the origin site of several clinically important diseases such as high-grade serous ovarian cancer (the deadliest form of gynecological cancer), pelvic inflammatory disease (PID), and infertility [19, 20]. Direct examination and study of fallopian tubes in alive patients is extremely difficult and somehow impossible due to its intra-abdominal location and structure. Therefore, an in vitro organoid model that recapitulates the in vivo structure and function of fallopian tubes is advantageous for supporting studies in this field.

**Fig. 1:**
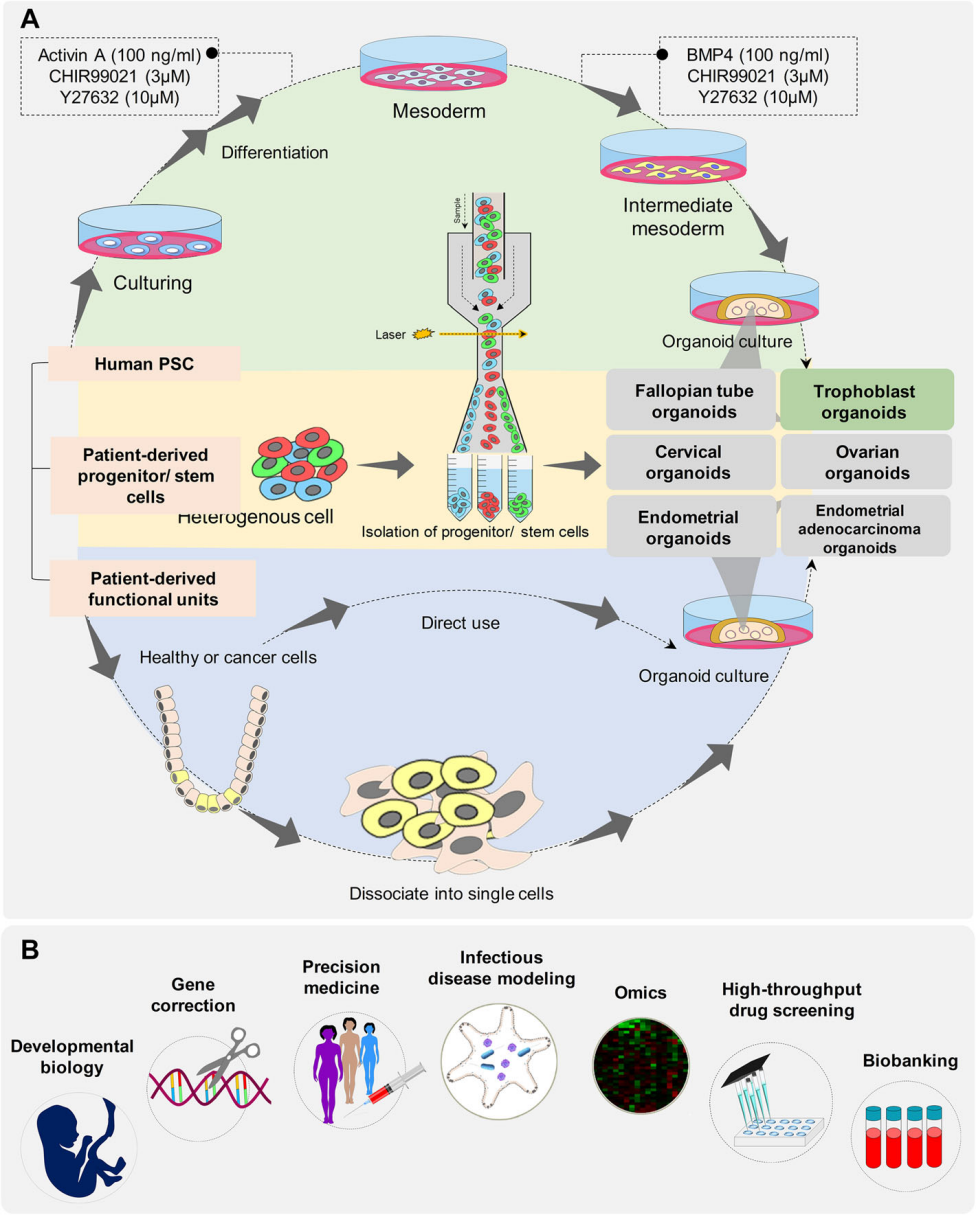
Schematics of mainstream human reproductive organoids and their applications. Different reproductive organoid development processes. Human reproductive organoids can be generated from normal or malignant primary tissues. Alternatively, somatic cells can be reprogrammed to become induced pluripotent stem cells, which are used as sources of reproductive organoids through directed differentiation. Primary tissues are dissociated into functional units that contain adult stem cells. These functional units can be digested into single cells and sorted to enrich stem cells for an organoid culture. Furthermore, iPSCs undergo directed differentiation towards the desired germ lineage and are subsequently embedded in matrix such as Matrigel to initiate an organoid culture. Organoids are typically cultured in an extracellular matrix (ECM) surrounded by culture media supplemented with specific niche factors. Applications of reproductive organoids. In basic research, organoid technologies provide new insights to understand the principles of development, homeostasis, and regeneration. Moreover, targeted gene therapy using the CRISPR/Cas9 system can be used on organoids derived from disease tissue. In personalized medicine, patient-derived organoids (PDO) can help to identify the best drug for each patient and diseases, including endometrial and ovarian cancers. Omics analysis (transcriptomics, proteomics, epigenomics, and metabolomics) of healthy and diseased organoids can reveal the molecular mechanisms involved in tissue differentiation and diseases. Bio-banked organoids can be used to identify drugs that are effective against a broad spectrum of disease phenotypes. Organoids also represent useful tools for the study of infectious diseases because they replicate the complexity of the in vivo system yet still retain the accessibility of an in vitro system

There are two main methods for fallopian tube organoids, the air-liquid interphase (ALI) system and self-organizing organoids. Chen et al. have applied an ALI system for long-term culture of murine, porcine, and bovine (three common species used in reproductive biology) oviductal epithelial cells (OEC). Isolated OEC have been cultured within an insert submerged in a proliferation-inducing medium for 7 days to form a confluent layer, and then the medium was aspirated from apical compartment to establish an air-liquid interface that supported polarization and differentiation. Subsequently, during the differentiation period, the cells were grown in serum-free or serum-reduced media from the basolateral compartment for two or 3 weeks. During this period, the cells developed from a flat layer to a columnar-shaped layer that consisted of ciliated and secretory cells. The polarized structure was maintained for at least 6 weeks. After the differentiation period, the cells secreted an oviduct fluid surrogate that could support embryonic development up to the blastocyst stage without the addition of embryo culture medium [21]. Despite the polarized structure and in vivo-like function, this culture system differs from the current organoid concept. This culture system lacks the tubular folded architecture and inserts, and does not permit live imaging or perfusion studies that limits its use to study gamete interactions and early embryo development in detail [8, 21]. In self-organizing organoids, the fallopian tube was generated based on the modified intestinal organoid protocol. Isolated fallopian epithelial cells were seeded in a 2D culture, followed by culture in 3D Matrigel matrix supplemented with growth factors (epidermal growth factor [EGF], fibroblast growth factor [FGF], and TGF-β), niche specific factors (Wnt3a, R-spondin-1 [RSPO1], ALK4/5, and Noggin), and an inhibitor of anoikis (ROCK inhibitor) (Fig. 1, Table 1) [25]. In contrast to other organoid models, this study showed that the addition of Wnt3a and RSPO1 maintained the stem cell subpopulations for an extended period of time and also allowed full differentiation. The presence of EGF doubled the number of organoids, addition of RSPO1 increased their size, and addition of ALK4/5 was crucial for quasi-indefinite expansion (Table 2). Monoclonal cystic organoids that contained ciliated and secretory cells have been successfully generated from a single EpCAM+ cell. Fallopian tube organoids faithfully recapitulate the structure of native tissue, show highly polarized columnar cells, fully developed inter-cellar junctions, fully assembled cilia, active secretion, and an orientation of the apical pole to the luminal side. These organoids are responsive to hormonal stimulation, show robust growth and can be maintained long-term in culture. Successful generation of monoclonal organoids from different donors has confirmed the presence of stem cells in the generated organoids, as well as fallopian tube epithelium (FTE).

**Table 1:** Frequently used growth media constituents, their working mechanisms, effects and applications

		Mechanism of action	Function	Significance in reproductive organoid cultures	Ref
Frequently used factors	Noggin	Binds and inactivates members of the TGF-β superfamily signaling proteins, such as BMP4.	Allows for long-term expansion of organoids by preventing differentiation.	The lack of Noggin resulted in reduced numbers and/or smaller human EMO. The lack of Noggin growth markedly reduced the expansion and passageability of mouse EMO. Limited differentiation and promoted trophoblast survival in CTB-orgs.	[3–5]
	RSPO1	Interacts with WNT4 in the process of female sex development. Potentiates the cellular response to Wnts.	Facilitates the growth, expansion, and long-term culture of organoids. Plays a crucial role in formation and stem cell maintenance of FTO.	The lack of RSPO1 resulted in reduced numbers and/or smaller human EMO. Removal of RSPO1 alone did not affect mouse EMO formation efficiency. The lack of RSPO1 markedly reduced growth, expansion and passageability of mouse EMO. RSPO1 was required for efficient, long-term human EMO expansion. In the absence of RSPO1, human organoids could no longer be passaged after P3. Addition of RSPO1 increased the FTO size. RSPO1 is not absolutely required for long-term maintenance of mouse FTE organoids. Addition of RSPO1 increased differentiation of FTE organoids toward the ciliated lineage. Withdrawal of RSPO1 promoted trophoblast outgrowth from the outer CTB layers and expression of HLA-G at distal sites from the CTB-orgs. Critical for maintenance of CTB-orgs.	[4, 5, 22, 23]
	WNT3A	The ligand of the canonical Wnt signaling pathway, Interacts with the LRP6/Frizzled receptor complex	Crucial for maintenance of stable growth over time.	The lack of WNT3A markedly reduced growth, expansion and passageability of mouse EMO. The presence of WNT3A (alone or together with RSPO1) enhanced the efficiency of mouse EMO formation. WNT3A was not needed for further expansion and passaging of human EMO.	[4]
	HGF	Activates a tyrosine kinase signaling cascade after binding to the proto-oncogene c-Met receptor	Epithelial-cell mitogen.	The lack of HGF resulted in reduced numbers and/or smaller human EMO. Limit differentiation and promote trophoblast survival in CTB-orgs	[4, 5]
	EGF	Activates the RAS/RAF/MEK/ERK signaling pathway	Epithelial-cell mitogen. Crucial for maintenance of stable growth over time. Supports proliferation and differentiation of FTE cells.	The lack of EGF resulted in reduced numbers and/or smaller human EMO. The lack of EGF resulted in markedly reduced growth, expansion, and passageability of mouse EMO. Required for long-term expansion of CTB-orgs.	[4, 5]
	FGF10	Acts mostly on the epithelium via Fgfr2b.	Epithelial-cell mitogens.		
	Prostaglandin E_2_	Binding and activation of the prostaglandin E2 receptor.		Critical for maintenance of CTB-orgs.	[5]
	Nicotinamide	A form of vitamin B3.		Withdrawal of nicotinamide had the strongest effect on the numbers of EMO that formed.	[3]
Molecule inhibitors	Y27632	Inhibits anoikis of dissociated cells.		Important for long-term culture and passaging of organoids.	
	A-83-01	Alk3/4/5 inhibitor. Blocks the TGF-β pathway.	Maintains epithelial-cell features	The lack of A-83-01 resulted in reduced numbers and/or smaller human EMO. Critical for maintenance of CTB-ORGs. Required for long-term expansion of CTB-orgs.	[4, 5]
	SB202190	p38 inhibitor.		Decreasing concentrations of p38i were beneficial for long-term expansion of endometrial cancer organoids.	[24]
	SB431542	TGF-β R kinase inhibitor IV.	Crucial for quasi-indefinite expansion of FTO.	Without TGF-β, RK inhibitor FTO had slower expansion and finally growth arrest by four to six passages (three to four months). Important for formation and maintenance of large FTE organoids.	[23, 25]
	CHIR99021	Inhibitor of GSK3.		Critical for maintenance of CTB-orgs.	[5]

*EMO* Endometrial organoid, *FTO* Fallopian tube organoid, *CTO* Cytotrophoblast, *BMP4* Bone morphogenetic protein-4, *RSPO1* R-spondin-1, *Fgfr2b* Fibroblast growth factor receptor 2b, *FTE* Fallopian tube epithelium, *HGF* Hepatocyte growth factor

**Table 2:** Summary of sources and culture conditions used in the development of various reproductive organoids

Organoids	Source	Culture conditions	Cell types in organoids	Matrix	Generation efficiency	Reference
Ovarian cancer	Ovarian cancer tissue	**Establishment**: Advanced DMEM/F12, Glutamax, HEPES, Noggin, Rspo1, WNT3A, B27, FGF10, bFGF, nicotinamide, N-acetyl-L-cysteine, A83–01, Heregulinβ-1, hEGF, IGF-1, HGF, Forskolin, Hydrocortisone, NRG1, p38i (SB203580), β-Estradiol, Y-27632 **Test components:** carboplatin, paclitaxel, alpelisib, pictilisib, MK2206, AZD8055, Niraparib, adavosertib, gemcitabine, doxorubicin, nutlin-3	Disease and original tumor phenotype	Cultrex growth factor reduced BME type 2	33–65%	[15, 16]
Fallopian tube	human iPSC lines (87iCTR-n3, 01iMEC-n4, 14iCTR-n6)	**Mesoderm induction:** Activin A, CHIR99021, Y27632 **Intermediate mesoderm induction:** BMP4, CHIR99021, Y27632 **The fallopian tube epithelial cells differentiation:** WNT4, Follistatin **Establishment:** DMEM/F12, reconstituted Ultroser, Y-27632, estrogen, progesterone, conditioned media from FTE cells **Maturation:** cultured in 3D Matrigel for an extended period with estrogen and progesterone supplemented media	Ciliated (TUBB4A and FOXJ1) cells secretory (PAX8) cells	Matrigel	NR	[22]
	Lin − EPCAM+ FTE cells	**Establishment:** Advanced DMEM/F12, GlutaMAX, B27, EGF, TGFBR1 Kinase Inhibitor IV, Y-27632 **Differentiation**: RSPO1	PAX8+ secretory cells acetylated tubulin (AcTUB) + ciliated cells	Matrigel	NR	[23]
	Epithelial progenitor (EpCAM+) cell	**Establishment:** Advanced DMEM/F12, conditioned human Wnt3A medium, conditioned human RSPO1 medium, GlutaMAX, B27, N2, human EGF, human noggin, human FGF10, nicotinamide, Y-27632, TGF-β R Kinase Inhibitor IV **hormonal stimulation:** beta-oestradiol, progesterone	Pax8-positive secretory cells Pax8 negative, acetylated tubulin-positive ciliated cells	Matrigel	NR	[25]
Endometrium	the mouse endometrial glandular-type fragments	**Establishment**: DMEM/F12, GlutaMAX, B27, ITS, FGF10, nicotinamide, WNT3A; R-spondin 1, Noggin, A83–01 **Hormonal stimulation:** beta-oestradiol, progesterone	secretory cells ciliated cells	Matrigel	NR	[4]
	the human endometrial glandular-type fragments	**Establishment**: WNT3A; R-spondin 1, EGF, FGF10, Noggin, A83–01, ITS, N-acetyl-L-cysteine, p38 inhibitor SB202190**Hormonal stimulation:** beta-oestradiol, progesterone	secretory cells ciliated cells	Matrigel	NR	[4]
	Dissociated endometrial cancer cells	**Establishment**: DMEM/F12, B27, Glutamax, N-acetyl cysteine, Primocin, nicotinamide, A 83–01, SB 202190, Y- 27632, 17-A estradiol **Test components:** Megestrol acetate, fulvestrant, letrozole, mifepristone, erlotinib, linsitinib; Selleckchem, BGJ-398, BBI608, cisplatin, paclitaxel.	Epithelial cells mesenchymal derivatives	growth factor reduced BME type 2	NR	[26]
	Endometrial epithelial cells	**Establishment: ExM:** Advanced DMEM/F12, N2, B27 minus vitamin A, Primocin, N-Acetyl-L-cysteine, L-glutamine, Recombinant human EGF, Recombinant human Noggin, Recombinant human Rspondin-1, Recombinant human FGF-10, Recombinant human HGF, ALK-4, −5, −7 inhibitor A83–01, Nicotinamide **Differentiation:** β-oestradiol, progesterone, cAMP, prolactin, human chorionic gonadotropin, human placental lactogen **Hormonal stimulation:** β-oestradiol, progesterone, cAMP	secretory (PAEP^+^) cells ciliated (acetylated-α-tubulin^+^) cells	Matrigel	100%	[3]
	Endometriotic epithelial cells (ECT-O) Epithelial cell from hyperplastic endometrium (HYP-O)	**Establishment; SOM;** DMEM/F12 + L-glutamine and Hepes, RSPO1-conditioned medium, Noggin, B27, N2, Glutamax, Insulin Transferrin Selenium, Nicotinamide, A83–01, N-acetyl L-cysteine, EGF, bFGF, FGF-10, SB202190 (p38i)	secretory cells ciliated cells	Matrigel	ECT-O; 60% HYP-O; 70%	[24]
	Epithelial cell from endometrial cancer	**Establishment; SOM;** DMEM/F12 + L-glutamine and Hepes, RSPO1-conditioned medium, Noggin, B27, N2, Glutamax, Insulin Transferrin Selenium, Nicotinamide, A83–01, N-acetyl L-cysteine, EGF, bFGF, FGF-10, insulin-like growth factor 1 (IGF1), hepatocyte growth factor (HGF) and lipids **Test components:** paclitaxel, 5-fluorouracil, carboplatin, doxorubicin and everolimus	NR	Matrigel	EC-O; 40%;	[24]
Trophoblast	villous cytotrophoblasts (vCTBs), purified from pooled first-trimester placental tissues	**Establishment:** basic trophoblast organoid medium (b-TOM): advanced DMEM/F12, HEPES, B27, N2, glutamine, R-spondin, A83–01, rhEGF, rmHGF, prostaglandin E2, CHIR99021, Noggin, EGF, R-spondin, CHIR99021, A83–01 **Differentiation**: lacking R-spondin and CHIR99021, inhibitor of Wnt response-1 (IWR-1)	cytotrophoblasts (CTB) syncytiotrophoblasts (STB) extravillous trophoblast (EVT)	Matrigel	100%	[5]
	trophoblast-enriched cell suspensions	**Establishment:** basal trophoblast organoid medium (TOM): EGF, FGF2, CHIR99021, A83–01, R-spondin 1, HGF, PGE2, Y-27632, nicotinamide**Differentiation**: EVT differentiation medium (EVTM): advanced DMEM/ F12, 2-mercaptoethanol, BSA, ITS-X, NRG1, A83–01, KSR. Typically after days 7–10, the medium was changed to EVTM without NRG1 for a further 7–10 days.	syncytiotrophoblast (SCT) villous cytotrophoblast (VCT) HLA-G^+^ extravillous trophoblast cells (EVT) cells	Matrigel	91%	[27]
Cervical organoid	cervical clear cell carcinoma cells	**Establishment:** DMEM/F12, human EGF, R-spondin1, Noggin, Y27632, Jagged-1, l-glutamine **Test components:** paclitaxel, cisplatin, gemcitabine hydrochloride, crizotinib, and SU11274	Atypical cells with clear cytoplasm concordant with morphological features of the original tumor	Matrigel		[28]

Abbreviations: *A.83–01* Selective inhibitor of ALK4,5,7, *EGF* Epidermal growth factor, *FGF* Fibroblast growth factor, *HGF* Hepatocyte growth factor, *NRG1* Neuregulin-1, *Rho-KI* Rho kinase inhibitor, *RSPO1* R-spondin-1, *NR* Not-reported

Scientists reported a human iPSC reprogramming method for generating FTE organoids. In this study, different WNT and BMP signaling were modulated to successful direct differentiation of human pluripotent stem cells into Müllerian cells and subsequent pro-Müllerian growth factors were used to develop FTE precursors. Then, FTE precursors were cultured in Matrigel with phenol red where they formed an organoid structure. However, when cultured in Matrigel without phenol red, they became branched and formed an unorganized matrix [22]. Phenol red is widely used in cell culture as a pH indicator; it bears structural similarity to nonsteroidal estrogens, exhibits estrogen-like bioactivity, and promotes proliferation in estrogen-sensitive cells such as fallopian tube cells [29, 30]. Therefore, their results have shown that estrogen effects FTE differentiation and maturation [22]. Human iPSC-derived FTE organoids were grown in 3D Matrigel with estrogen and progesterone supplemented media for an extended period. Immunocytochemistry results showed that FTE organoids formed secretory (PAX8+) and ciliated (TUBB4A+) cells. Expression of a mature epithelial cell marker (CDH1) in the organoid was comparable to fresh human fallopian tube tissue. In addition, the proper differentiation of iPSC-derived organoids into fallopian tube cells was confirmed using heat map analysis [22].

The described fallopian tube organoid models closely mimic normal physiology and architecture of the human FTE. Therefore, they provide promising models to study the biology and pathology of fallopian tubes with regards to screening technologies, cancer biology, and reproductive medicine [25]. However, this system has limitations for gamete or embryo interaction studies due to its small size and inaccessible luminal compartment that require labor-intensive approaches, such as microinjection.

### Endometrial organoids

The human endometrium is a dynamic tissue that undergoes cyclic changes in response to steroid hormones as well as paracrine and autocrine factors to be prepared for embryo implantation. Embryo implantation is a highly complex process that requires a receptive endometrium, a competent blastocyst, and a synchronized maternal-embryo dialogue [31]. The endometrium is also involved in many gynecologic conditions, including infertility, dysmenorrhea, endometrial polyps, endometriosis, and endometrial cancer which is the most common cancer of the female reproductive organs [32].

For first time, Bläuer et al. developed and validated a culture condition in which normal human endometrium was cultivated as glandular organoids within Matrigel matrix in co-culture with stromal cells. However, this 3D culture system differed in principle and protocols from the currently adopted organoid concept [33]. Successful generation of endometrial organoids was reported by two separate groups in 2017 for mouse and human endometria [3, 4]. These endometrial organoids were established by embedded dissociated endometrial cells in Matrigel droplets in culture medium (Fig. 1 and Table 2) that are commonly used to support the development organoid models of other organs. The endometrial organoids recapitulated the molecular and functional characteristics of their cells of origin. Endometrial organoids, like in vivo endometrium, exhibit glandular-type self-organization, apicobasal polarity, and functional behavior such as mucus production, and are responsive to sex hormones [3, 4]. Endometrial organoids have been derived from endometrial adenocarcinomas and the normal adjacent endometrium from post-menopausal women [3]. Unlike healthy endometrial-derived organoids, the tumor-derived organoids presented with a range of patient-specific morphologies, including pleomorphic cells with hyperchromatic nuclei, disorganized epithelium, basement membrane breaching and invasion of isolated cells into the surrounding Matrigel. However, the tumor derived-organoids were positive for glandular markers MUC1 and SOX17, which confirmed their glandular origin. Investigation of chromosomal stability of endometrial organoids using a comparative genomic hybridization (CGH) array demonstrated that the established organoids preserved their genetic integrity over several months in culture. These genetically stable endometrial organoids could be expanded and frozen without loss of their proliferative ability after thawing to create a patient-specific biobank of endometrial tissues [3]. This culture system has the ability to expand the small quantity of starting material for a variety of high throughput assessments and could be a valuable platform for investigating implantation problems, the histotrophic nutrition period in early pregnancy, novel therapeutic strategies for gynecologic pathologies such as endometriosis and endometrial cancer, and generate an endometrial biobank. However, the current organoids lack stromal cells. It is well known that reciprocal interaction between endometrial epithelial and stromal cells is responsible for physiological functions (proliferation, differentiation, and decidualization) and emergence of several pathologic conditions such as endometrial carcinoma [34–36]. Without a more complete complement of cell types, endometrial organoids will always lack the context they need to be actual mini-organs. Recently, a co-culture of intestinal organoids with stromal cells was developed by Stzepourginski et al. [37]. Co-culture of the present endometrial organoids with stromal cells could further complement the organoid model and provide a relevant model to study reciprocal epithelium stroma interactions that occur in vivo [36]. The inaccessible luminal compartment and apical aspect of the epithelium of organoids present a number of challenges to physiologically relevant studies [38]. Unfolding the spherical organoid into a 2D planar tissue construct and monolayer culture of primary epithelial cells from organoids would provide an accessible luminal compartment for related studies, such as embryo cultures [38].

### Trophoblast organoids

The placenta is an extraembryonic organ that is essential for survival and development of the mammalian embryo. During implantation in humans, the trophectoderm layer of the blastocyst attaches to the endometrial epithelium and continues to differentiate into trophoblast subtypes: the cytotrophoblast (CTB), extravillous CTB (EVT), and syncytiotrophoblast (STB). Undifferentiated CTB cells grow through the STB to form cell columns and chorionic villi. The CTB cells at the tips of villi differentiate into EVT, which invade the deciduum. EVTs have two cell types: interstitial EVT cells that invade the decidualized endometrium and endovascular EVTs that invade and remodel the spiral arteries [39, 40]. Multinucleated STB cells are responsible for nutrient exchange and synthesis. They secrete placental hormones such as placental lactogen, chorionic gonadotropin, and progesterone in addition to numerous other proteins and steroids to maintain pregnancy. These cells are formed by fusion of interstitial CTB cells [41]. Placental dysfunction and insufficiency results in major pregnancy-related disorders such as intrauterine growth restriction, preeclampsia, miscarriage, recurrent abortion, and preterm labor [42, 43]. Our current knowledge about the human placenta is limited due to the lack of representative functional models [44]. Several experimental models, including animal models and in vitro cell culture models, have been used to study the placenta. However, distinct structural and functional differences exist between human and other animal placentas; thus, data from animal models is not relevant to humans [45, 46]. In vitro models of human trophoblasts include placenta-derived cell lines, isolated primary placenta cells, and human placenta tissue explants. Several cell lines have been established from choriocarcinoma cells: JEG-3, BeWo, and JAR. Advantages of these cell lines include ease of use; unlimited supply of material; more pure cell source; less expensive to procure; ease of use for gene silencing approaches; and these sources bypass some of the ethical concerns associated with the use of animal or primary human tissues [47]. However, immortal cell lines have several drawbacks due to their malignant transformations. This problem may be overcome by using immortalized primary trophoblast cells, such as the HTR-8/SVneo cell line derived by transfecting first trimester EVT cells with the gene that encodes for simian virus 40 large T antigen, or ACH-3P and AC1-M59 cell lines, which are choriocarcinoma cells fused with primary first and third trimester trophoblast cells, respectively [48]. However, some of these cells do not meet the criteria for human trophoblast cells as proposed by Lee et al., and do not express some important protein markers (GATA3, KRT7, and TFAPC2), the human leukocyte antigen (HLA) class I profile, the chromosome 19 miRNA cluster (C19MC), and hypomethylation of the ELF5 promoter [49]. Alternative models, such as bone morphogenetic protein 4 (BMP4)-treated human embryonic stem cells (hESCs), have also been established. Although these trophoblast-like cells are used as models for understanding early trophoblast lineage segregation, their global gene expression profiles, trophoblast-specific markers, and HLA status considerably differ from primary trophoblast populations [50].

**Fig. 2:**
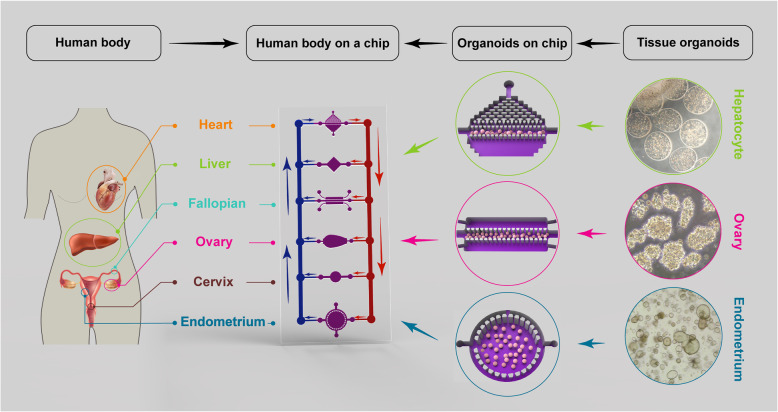
The multi-organoid-on-a-chip system containing the female reproductive, the liver and the heart organoids. Culture medium can be circulated within each organoids, between organoids, and within the entire system, enabling the controlled and biomimetic distribution of biomolecules, such as metabolites and hormones. In the multi-organoid-on-a-chip systems, the responses of one organoid to drugs or toxins affect the responses of other organoids, which occurs in actual human physiology. In addition, these micro-physiological systems can use to study the effects of reproductive hormones throughout the body. The phase contrast images of organoids in the right part of figure is from correspond author’s lab (un-published data). Heart image in female body has been adapted from Freepik.com designed by Kjpargeter; Liver and uterus images in female body have been adapted from Freepik.com designed by macrovector (These three elements have been adapted from resources of Freepik.com in 27 may 2020). Other elements in the figure have been designed by authors

Recently, two independent research groups developed organoid platforms for culturing first-trimester CTBs in vitro*. Turco* et al. *reported some growth cell clusters of* first-trimester placenta were seeded into Matrigel drops and grown in a basal trophoblast organoid medium (TOM) composed of EGF, FGF2, CHIR99021, A83–01, and RSPO1 (Table 1, Fig. 1). Addition of growth factors hepatocyte growth factor (HGF), PGE2, and Y-27632 increased cell viability and growth. In combination with TOM, the researchers observed rapid expansion of cells within a week (Fig. 2) [27]. Haider et al. *showed that when villous CTB (vCTBs) were embedded in Matrigel that contained a defined cocktail of growth factors and signaling inhibitors, small cell clusters formed within several days of culture. These clusters rapidly grew and developed into organoids with irregular structures after 2–3 weeks* [5]*.* Under these two well-defined conditions, organoid structures appeared and homogeneous trophoblast organoids were established within 10–14 days (two passages). To confirm the fetal origin of trophoblast organoids, they used microsatellite analysis and HLA typing. The generated trophoblast organoids were genetically stable after consecutive passages for more than 6 months and had mitochondrial function after cryopreservation. Finally, they provided evidence that the organoids were of clonal origin and that large organoids were formed from single cells within 3–4 weeks [5]. Identities of these trophoblast organoids were verified against trophoblast-specific criteria at similar or higher levels than the choriocarcinoma lines JEG-3 and JAR [27]. Principal component (PC) analysis of placental villi, trophoblast organoids, placental stromal cells, and decidual organoids based on 12,673 probes showed that the trophoblast organoid clusters were more closely related to the placenta with enrichment for trophoblast-specific genes such as *CGB3*, *GATA3*, and *PSG6* [27]. The trophoblast organoid structures closely recapitulated the organization of placental villi in vivo, including the presence of a surrounding basement membrane beneath the VCT, SCT in the center of the organoids, syncytial masses which line the central cavity, abundant secretory organelles, and surface microvilli (confirmed by electron microscopy) [27]. The secretory function of trophoblast organoids was confirmed using proteomic analysis of the conditioned medium by liquid chromatography-tandem mass spectrometry (LC-MS/MS). Trophoblast organoids secrete a wide range of placental-specific peptides, hormones and enzymes, including pregnancy-specific glycoprotein (PSG), early placental insulin-like protein (INSL4 or EPIL), human chorionic gonadotropin (hCG), growth differentiation factor 15 (GDF15), kisspeptin (KISS1), chorionic somatomammotropin hormone 1 (CSH1), and aldose reductase [27]. HLA-G, a potent tolerogenic molecule at the maternal-fetal interface, is highly expressed by both endovascular and interstitial EVT and increases during trophoblast migration towards the spiral arteries [51]. In basic organoid medium, a few HLA-G^+^ cells were found in trophoblast organoids, but under an EVT differentiation protocol suggested by Okae et al. [52] and by using EVT differentiation medium (EVTM). HLA-G^+^ cells that migrated out of the organoids appeared and invaded in the 3D culture, and adhered to the plastic dish. Hence, the organoids mimicked the villous placenta in an anatomic, functional, metabolic, and endocrinologic manner [5, 27].

Cumulatively, it is suggested that the trophoblast organoid model is a promising tool to study human placental development and investigate trophoblast dysfunction and insufficiency. Developing an advanced culture system that contains both endometrial and trophoblast organoids would allow researchers to study the mechanisms that underlie maternal-fetal interactions during pregnancy.

### Female reproductive organoid applications

#### Drug discovery and toxicology study

The discovery and development of a new drug is a complex, time-consuming, and costly process accompanied by a high rate of failure [53]. Despite substantial progress made in pharmaceutical research in recent years, only a single drug among thousands of laboratory tested compounds reach the marketplace [54]. The main reason for this high rate of failure is related to the lack of reliable disease and relevant human models, and inaccurate results from animal models [55–57]. The developmental and reproductive toxicity of a vast majority of about 75,000–85,000 chemical substances in commerce has not been investigated [1]. Despite public concerns regarding the reproductive toxicity of chemicals, the field of reproductive toxicology (repro-toxicology) is in its infancy; therefore, more well-targeted research in this field is needed to better understand and prevent reproductive health risks. New approaches in medicine (precision medicine) focus on individual variations that lead to different patient responses for the same drug [58].

Although animal models are frequently used for drug screening and toxicity studies [59], they are not suitable. There is a need to confirm drug safety in both male and female humans [60] along with high-throughput screening of a wide range of drugs and compounds for different purposes, including development of novel contraceptive agents and vaccines, drug screening for infectious diseases, cancer drug development, gestational drug development and reproductive toxicity testing of drugs and compounds [61, 62]. Although traditional in vitro models such as the 2D monolayer cell culture provide valuable tools for drug screening and toxicity testing and have the potential to identify drug candidates, many challenges still remain. One of the main challenges is the change in cellular responses in these model systems that contributes to their unnatural microenvironment [63]. Conventional models, including animal models and traditional culture systems, are unable to provide a platform for precision medicine. Limitations associated with these models have encouraged development and validation of new in vitro models that mimic the complex and dynamic biological features of human tissues, re-create the function and structure of these tissues, and recapitulate in vivo physiology. In this regard, organoids are 3D miniaturizations of human tissues that exhibit native tissue architecture [64] that carries out person-specific genomic and epigenetic information. Organoids provide several unique advantages for drug screening and toxicology studies. These structures are frequently derived from primary cells. They consist of multiple cell populations, possess stable genotypes, and their capacity for self-renewal facilitates their propagation and expansion for drug screening and toxicity studies [65–67]. Since the use of organoids in reproductive medicine for toxicology studies, drug development and personalized medicine is still a naïve field, more studies are required to evaluate their potential as model systems.

An impressive proof-of-concept has been reported for organoid technology for drug testing in cystic fibrosis (CF) [68, 69]. CF is a life-shortening disease caused by loss of function mutations in the CF transmembrane conductance regulator (*CFTR*) gene, which results in an accumulation of abnormally viscous, sticky mucus in the gastrointestinal and respiratory tracts [70]. Females with CF have reduced fertility due to abnormally thick, dense cervical mucus, altered ion and fluid transport throughout the reproductive tract, and endocrine abnormalities or menstrual irregularities [70]. Over the past decade, high-throughput screening has led to the identification of small molecules that restore the defective protein and ameliorate the trafficking defects (correctors) and/or augment its activity (potentiators). However, CFTR-restoring pharmacotherapy has varying degrees of efficacy in treating CF, which suggests a contribution by more factors beyond the genetic background among individuals. Dekkers et al. have introduced a novel functional CFTR assay that used organoid cultures from rectal biopsies. They showed that forskolin induced swelling in the healthy organoids. Although this effect was strongly reduced in organoids established from CF patients, it was reversed by treatment with CFTR-restoring compounds [69]. Results of follow-up studies showed that in vitro drug responses in biopsy-derived organoids positively correlated with the clinical response to therapy [68, 71]. Hence, this platform could be used for preclinical selection of responders to pre-existent CFTR modulators as well as for the identification of novel CFTR-restoring compounds. Furthermore, in a recent study, endometrial cancer organoids exhibited patient-specific drug responses. A specific endometrial cancer organoid line was most sensitive to everolimus (an inhibitor of mammalian target of rapamycin [mTOR]), which suggested a strong dependence on the PI3K-AKT pathway and was in line with mutations in the pathway’s signaling mediators (*PTEN*, *PIK3CA*, *AKT1*) [24]. These evidences have shown that organoids are amenable to toxicology studies, drug development, and personalized medicine.

#### Organoid applications in studying reproductive infectious diseases

There are three types of human reproductive tract infections (RTIs) - STDs such as chlamydia, gonorrhea, syphilis, genital herpes, and human immunodeficiency virus (HIV); endogenous infections, which result from overgrowth of organisms normally present in a healthy women’s genital tract; and iatrogenic infections, which are associated with medical procedures such as induced abortions, poor delivery practices or intrauterine device (IUD) insertions [72, 73]. RTIs, generally seen as a ‘silent’ epidemic, are associated with complications of gynecologic and reproductive health, including endometritis, infertility, ectopic pregnancy, PID, chronic pelvic pain, miscarriage, and neonatal blindness. STIs/RTIs also increase the risk of HIV infection and can cause death [74–76]. The morbidity associated with RTIs affects economic productivity and the quality of life of many individuals and, ultimately, entire communities. Inaccessibility of the internal organs for analysis of the long-term consequences of acute and chronic consequences of RTIs/STDs infections in vivo have encouraged the use of many experimental models such as in vitro, ex vivo, and animal models to study pathogenesis mechanisms, discover biomarkers, and perform drug candidate screenings [77–80]. Classically, because of the limited life span of primary cell cultures, the majority of studies have been conducted in immortalized cell lines, including HEC-1A cells [9], transformed endometrial line HEC-1B [81–83], Ishikawa cell line [83], uterine epithelial cell (UECs) line ECC-1 [84], OE-E6/E7 (OEC line) [85, 86], the transformed epithelial cervical line HeLa [87], and HEp-2 epithelial cell line [88, 89]. These cell lines are relatively easy to maintain and have provided important insights into understanding host-pathogen interactions. However, the lack of complexity of these cell types, failure to recapitulate the architecture of in vivo tissues, and inability to produce some tissue-specific factors are main limitations for their use. Thus far, several 3D cell culture systems of human reproductive tissues have been developed that have the potential for use in infectious disease research. These 3D cell culture systems include the rotating wall vessel (RWV) bioreactor [90–92], organoids [3, 4, 23, 25], and organ-on-a-chip (OOAC) [21] models. A major challenge in infectious disease modeling is the recreation of a 3D microenvironment of native tissues to more accurately model the initiation and progression of an infection. Long et al. [93] and Nickerson et al. [94] have reported the first studies that investigated viral and bacterial infections in 3D models, respectively. In recent years, 3D models have gained increasing interest in the field of infectious disease because of their potential to enhance understanding of disease pathogenesis and in drug screening.

Since organoids exhibit enhanced in vivo-like features, including apico-basal polarization, appropriate localization of cell adhesion molecules, cytokine production, responses to antimicrobials and microbial products, support of commensals, and/or susceptibility to infection, they are theoretically well-suited for infectious disease studies. Various pathogens that have been studied using organoids include Helicobacter pylori (stomach organoids) [95, 96], *Salmonella enterica* [97] and *Clostridium difficile* [98] in intestinal organoids, and Zika virus (ZIKV) in human neurospheres and brain organoids [99]. Kessler et al. used human fallopian tube organoids and genital *Chlamydia trachomatis (C. trachomatis)* serovars D, K, and E for long-term in vitro infection analysis and investigated its effect on epithelial homeostasis [100]. The epithelial organoids responded to the infection with a fast, dynamic extrusion of intact Ctr inclusions and/or infected cells into the lumen, and with compensatory cellular proliferation that demonstrated a role for epithelial cells in the defense against this pathogen. Furthermore, the acute infection led to activation of multiple paracrine growth factors (TGF-β1, EGF, FGF). LIF signaling is a key player in the maintenance of stemness in the organoids. The gradual decrease in the number of ciliated cells and increase in CD24 + Epcam+ and CD133 + cells suggests a sustained shift in the regulation of epithelial renewal during an infection. Infected organoids have a less differentiated phenotype with higher stemness potential. Moreover, their methylation data support a potential role for chronic *C. trachomatis* infection as an epigenetic modulator. *C. trachomatis* increases hypermethylation of DNA, which is an indicator of accelerated molecular aging [100]. This organoid approach could contribute to a better understanding of the long-term effects of *C. trachomatis* infections in the development of tubal pathologies, including the initiation of high-grade serous ovarian cancer.

Along with other infectious models, organoids also have limitations; the site of infection in most pathogens is the apical portion of the epithelium and delivery of the pathogen to the lumen of the cystic organoids is a challenge. To circumvent this limitation, microinjections have been used to deliver pathogens to the luminal side of the organoids.

#### Organoid platform for studying reproductive cancers

Organoid technology provided a research platform to study reproductive cancers, including ovarian and uterine cancers. Ovarian cancer is the deadliest gynecologic cancer worldwide with approximately a 35% five-year survival rate [101]. It is reported that epithelial ovarian cancer (EOC) accounts for 85–90% of ovarian cancers and high-grade serous carcinoma (HGSC) represents nearly 70% of all EOCs, which is associated with a poorer prognosis [101]. The majority of HGSCs appear to arise from the secretory cells of the FTE rather than from the ovarian surface epithelium [102, 103]. A better understanding of the HGSC biology, FTE transformation, and initiation and progression of HGSC using relevant in vitro human models can lead to new-targeted therapies and immunotherapeutic approaches. Recently, an iPSC- and patient-derived human FTE organoid in vitro model with the relevant cell types (ciliated and secretory) of the human fallopian tube and luminal architecture that closely mimic the tissue-specific structure has been established [22, 25]. iPSC-derived organoids can help to understand physiological and pathological processes of fallopian tubes, provide a powerful platform for drug screening, and develop novel therapies. This in vitro model can provide an appropriate model to explore the fallopian tube origin of HGSC, investigate early processes in the initiation and progression of HGSC, and study germline mutations and genetic alterations involved in HGSC [25]. While iPSC-derived FTE organoids exhibit many features of in vivo tissue, their application in high-throughput screening remains difficult due to limited culture scalability. Over the past decade, numerous literatures in the field of ovarian cancer support an oncogenic role of Notch signaling in HGSC. Notch signaling may be involved in ovarian cancer initiation, progression, metastasis, resistance to chemotherapy, cancer stem cell activity, angiogenesis, and epithelial-to-mesenchymal transition (EMT) [104]. Kessler et al. have reported that Notch signaling is a crucial regulator of differentiation and stemness in fallopian tube organoids; inhibition of the Notch pathway by addition of DBZ (Notch γ-secretase inhibitor) changes the differentiation pattern and structure of these organoids. Their results showed that 78 of the 274 ‘stem cell signature genes’ were also significantly downregulated in the DBZ-treated organoids. This fallopian tube organoid along with the above findings enabled researchers to better understand tubal epithelium pathology, including its role in ovarian cancer because most models of tubal carcinogenesis postulate that secretory cell outgrowth is the initial step toward malignant transformation [25, 105, 106]. Potential therapeutic targeting of the molecular aberrations and cellular signaling pathways involved in initiation and progression of ovarian cancer using organoid technology may provide novel treatment options for cancer patients [104]. The ability to efficiently introduce specific genetic alterations to epithelial precursor cells or human iPSCs and generate genetically engineered organoids using the CRISPR/Cas9 system will enable researchers to gain further insight in cancer research.

Uterine malignancies represent the most common diagnosed gynecologic malignancy worldwide and the fourth most common malignancy in women [107]. About 95% of these malignancies are endometrial carcinomas, the origin of which belongs to the endometrial glandular epithelium. The mesenchymal component such as endometrial stromal sarcoma or mixed epithelial and stromal tumors comprise the remainder [108]. The common treatment strategy for endometrial cancers includes surgery (hysterectomy with bilateral salpingo-oophorectomy) followed by chemotherapy and sometimes radiation therapy. If fertility is desired, hormonal therapy may be administered. However, in two meta-analyses that reviewed trials of recurrent or advanced endometrial cancer and trials on progestin in the adjuvant setting, found no evidence of substantial benefits from these drugs [109, 110]. During the past decade, our knowledge of the genetic basis for endometrial cancers has increased exponentially. Data from the Cancer Genome Atlas (TCGA) project showed that 373 patients with endometrial carcinomas had frequent mutations in PTEN, CTNNB1, PIK3CA, ARID1A, PPP2R1A, KRAS, MYC, ERBB2, CTNNB1, CCNE1, FGFR3,S OX17, TP53, PTEN, ARID5B, PIK3R1, FBXW7, and POLE [111]. Today, treatment of endometrial cancers is not based on patients’ genetic characteristics because of differences in mutations between patients. Therefore, personalized medicine that integrates data from whole genome sequencing (WGS) and whole exome sequencing (WES) to identify patients with specific cancer-related mutations with the drug screening patient-derived organoid (PDO) models can provide a platform for developing an effective therapeutic strategy for cancer patients. Successful treatment of endometrial carcinoma will require individualization of therapies based on the molecular and/or genetic make-up of the endometrial carcinoma cells. PDO cultures hold promise as an in vitro model for accurate recapitulation of a wide variety of normal and oncogenic in vivo cellular behaviors. To date, 3D organoids have been generated from biopsies and/or surgical resections of cancers of the colon [112], pancreas [113], lung [114], and prostate [115]. After the establishment of human endometrium organoid culture protocols, Turco et al. [3], Girda et al. [26], Pauli et al. [116], Dasari et al. [117], and Boretto et al. [24] have reported that it is feasible to grow organoids from primary endometrial cancer.

Organoids derived from endometrial cancer patients recapitulate the morphology of the primary tumors and show similar immunohistochemistry (IHC) characteristics with the primary tumors. IHC for MUC1 and SOX17 on both tumor and normal organoids has confirmed their glandular origin [3, 26]. The effects of verteporfin (VP), an FDA approved light-activated drug used in photodynamic therapy (PDT) for macular degeneration, was tested on an organoid model generated from tumor cells of type 1 endometrial carcinoma patient tissue [117]. VP-treated organoids had less expression of YAP and phospho-YAP, and higher expression of cleaved caspase-3. This finding suggested that VP induces apoptosis and more inactive YAP in the cells of organoids [117]. A high throughput drug screen that used a comprehensive library of up-to-date targeted agents was performed on uterine carcinosarcoma and endometrial adenocarcinoma organoids derived from patients with similar driver mutations in PIK3CA and PTEN. The results showed an association between genotype and drug response profiles. For the endometrial adenocarcinoma organoids, combined treatment with buparlisib (PI3K inhibitor) and olaparib (PARP inhibitor) was optimal when compared with other combination strategies. In contrast, for the endometrial adenocarcinoma organoid, the combination of buparlisib and vorinostat (HDAC inhibitor) was among the most effective treatments [116]. Results of a recent study of organoids from endometrial diseases indicated that the organoids were created from different grades and progression stages of endometrial cancer with lower efficiency compared to other endometrial conditions (20% for endometrial cancer organoids versus 100% for eutopic endometrial organoids and 70% for hyperplastic endometrial [HYP-O] organoids). An optimized culture condition by reducing the p38i concentration and adding insulin-like growth factor 1 (IGF1), HGF, and lipids enhanced the efficiency of organoids generated from endometrial cancers. Endometrial cancer organoids have morphological heterogeneity. Those derived from low-grade/stage cancers exhibit glandular-like morphology with a defined lumen. However, endometrial cancer organoids derived from high-grade/stage cancers commonly appear to be dense and lack a visible lumen [24]. Microsatellite instability (MSI) involved in the pathogenesis of about 30% of endometrial cancer cases [118] was also observed in the endometrial cancer organoids [24]. CGH array or low-coverage WGS revealed that the large majority of no somatic copy number alteration (SCNA) in primary tumors were retained in the corresponding cancer organoids, and the majority of the genetic alterations in the primary tumors were retained in the organoids after long-term expansion. Interestingly, a considerable number of new substitutions were retrieved in cancer organoids after long-term expansion [24]. Endometrial cancer organoids recapitulated the disease phenotype in vivo*.* Subcutaneously injected cancer organoids generated a cell mass that recapitulated histological and molecular features of the primary tumor. Orthotopic engraftment of high-grade cancer organoids into the uterine horn generated a large, invasive, and highly proliferative mass that had the potential for peritoneal metastasis [24]. These endometrial cancer organoids offer researchers a way to probe cellular pathways involved in tumorigenesis and provide a unique alternative model for endocrine profile studies, drug sensitivity, and for correlating data with the genetic landscape of individual tumors prior to treatment in humans. Clinical studies are needed to correlate organoid assay results with patient outcomes.

Organoids have been generated from young women with cervical clear cell carcinoma (cCCC), an extremely rare subtype of cervical cancer. The optimized protocol for organoid generation from gynecological tumors was applied to produce cCCC organoids. cCCC organoids were expanded in the laboratory setting by a modified Matrigel bilayer organoid culture. These organoids were successfully cryopreserved and recovered after thawing. A few mutations were identified in cCCC organoids and CCC component following genomic analysis. Two of these mutations were detected both in cCCC organoids and CCC component. Moreover, development of a xenograft was confirmed following cCCC organoid transplantation into nude mice. Spheroids derived from cCCC organoids showed drug sensitivity and proliferative capacity upon exposure to the anti-cancer drugs commonly used for gynecological cancer (paclitaxel, cisplatin, and gemcitabine) [28].

However, additional studies are needed to clearly emphasize genomic heterogeneity and transcriptome patterns between organoids and their tumors of origin before replacing the exciting models with organoids, implement cancer-derived organoids for high-throughput preclinical screenings, and design targeted and personalized therapies.

#### Organoids to study gynecological diseases

Endometriosis is a gynecological disorder that affects 10–15% of women worldwide and 30–50% of women with infertility. The disease is characterized by the growth of ectopic endometrial tissue outside the uterus [119]. Recently, the organoids from endometriosis patients in various disease stages (I, II, III, and IV) from both eutopic and ectopic origins have been developed [24]. These organoids displayed genomic stability during long-term expansion in culture and showed the same hormonal receptor expression as the original tissue. Endometriosis organoids injected into the peritoneal cavity generated implants that expressed endometriosis markers [24]. However, the decreased efficiency of organoid generation from endometriosis samples compared to healthy endometrial biopsies is a challenge. Moreover, the ability of endometriosis organoids to respond to hormones has yet to be addressed.

### Future therapeutic applications of endometrial organoids

Patients with Asherman’s syndrome (AS), intrauterine adhesions (IUA), thin dysfunctional endometrium, genital tuberculosis, repeated implantation failure (RIF), and endometrial atrophy (EA) resistant to hormonal treatment are candidates for endometrial reconstruction [120]. AS is an uncommon gynecological disorder associated with infertility, amenorrhea, hypomenorrhea, recurrent pregnancy loss, and abnormal placentation [121]. The current therapeutic approaches for AS are limited to surgical restoration and hormonal therapy [122] Surgical complications, serious side effects of hormonal therapy, and poor pregnancy outcomes in untreated AS cases indicate the need for new, safe, and effective treatment options [123, 124]. Stem cell therapy is a possible solution that offers a promising AS and EA treatment with the ultimate goal of replenishing the cellular compartments of the endometrium [121, 124, 125]. The successful use of stem cells to improve endometrial function and structure has been reported by several studies [126–129]. For successful stem cell therapy, transplanted cells must undergo several critical steps -proliferation, differentiation to tissue specific cell types, migration, distribution into an accurate location, and integration into the target tissue. In this process, a large number of stem cells may be eliminated from the chain of distribution, leaving only a few that survive and remain active. Expansion of stem cells without differentiation and genetic alteration is one of the main limitations of stem cell therapy [130]. The true value of a cultured cell as a candidate for cell-based therapy depends on its fidelity and expansion capacity, as well as its ability to maintain a normal genetic and epigenetic status [131]. Another obstacle is the lack of control over transplanted cells.

With the emergence of organoid technology, there is an increasing interest in the use of organoids for regenerative medicine. Advantages of organoids include their ability to be created from a small biopsy sample, massive expansion in vitro*,* and genetic and phenotypic stability over a long-term culture [3, 25]. Organoids have the potential to differentiate to a complete set of cell types of native tissue and provide high amounts of specific cell types for transplantation. This potential of organoids makes them different from traditional stem cell therapy, which uses specific stem/progenitor cells [3, 4, 25]. The successful engraftment of organoids has been previously reported. Results from an initial study of organoid transplantation reported that colon epithelial organoids were successfully engrafted in acute murine colitis model and covered the injured area shortly after transplantation. After a few weeks, this area contained proliferating cells and all colon specific cell-types. Transplanted organoids contributed to the normal epithelium for more than 6 months [132]. Upon transplantation, organoids have an intrinsic ability for self-renewal and self-organization, and can integrate into host tissue. Thus, stem cells or progenitor cells in organoids that have a higher survival rate and functional connections with the surrounding tissue in the host open a new and promising window for regenerative medicine [133]. Engraftment of endometrial organoids established from endometrium of TdTomato reporter mice under the kidney capsule of ovariectomized immunodeficient NOD/SCID/IL2Rgamma^null^ (NSG) mice [4] led to the generation of an organized structure with glandular-type protuberances [4]. Engrafted TdTomato^+^ endometrial organoids survived in vivo after 6 weeks, expanded, and assembled into organized glandular-type structures in response to E2 and Prog treatment [4].

Taken together, these studies show that transplanted organoids have the ability to integrate into host tissue, maintain their self-renewal and self-organizing ability, and differentiate to functional tissue-specific cells. Although organoid technology is currently in its infancy, organoids provide a promising platform for harnessing stem cell for regenerative medicine and healing damaged epithelia of specific diseases. The successful engraftment of endometrial organoids and their ability to survive in vivo and differentiate to endometrial specific cells suggests that endometrial organoid transplantation could be a novel approach to treat disorders related to an inadequate endometrium. In addition, autologous transplantation can overcome the traditional hazards associated with allogeneic transplantation.

### Reproductive system-on-a-chip (repro-on-a-chip): balancing sex differences in preclinical research

In the past decades, several studies have demonstrated that efficacy and safety of drug treatments are sex-specific and the female sex tends to have a higher risk of developing adverse drug reactions (ADRs) compared with the male sex [134, 135]. The reproductive system is comprised of sex organs that work together and produce various factors and hormones. These factors are not only necessary for reproduction and regulation of gamete production, but they also affect various organs and tissues, including the cardiovascular system, immune system, gastrointestinal system, nervous system, and liver [136]. Reproductive hormones and factors underlie sex-based differences in disease pathogenesis and development, and alter pharmacokinetics and responses to therapeutic agents [136]. One of the molecular mechanisms responsible for sex differences in drug metabolism may be due to differences in hepatic gene expression, especially those regulated by sex hormones (such as CYP3A4) [137]. CYP3A4, the major human drug-metabolizing cytochrome P450 (CYP) is expressed at higher levels in females [138]. Many drugs that are substrates of CYP3A4 such as cyclosporine, erythromycin, and nifedipine have a higher rate of clearance in females than males [137]. In the liver, sexual dimorphism in hepatic gene expression such as P450s is regulated by the plasma growth hormone (GH) that is synthesized by the pituitary gland [137]. The GH release pattern is pulsatile in males and almost continuous in females [139]. Reproductive steroid hormones can modulate GH action by regulating GH secretion from the pituitary glands and peripherally modulate GH signaling pathways through the GH receptor (GHR) [140]. In addition, a proteomic analysis on total liver protein extracts from male and female rats has demonstrated that liver protein expression is more affected by gender than by nutritional status [141]. There is a paucity of data on sex differences in preclinical and biomedical research and drug development. Traditional in vitro systems such as a monolayer culture of cell lines or primary cells without considering their sex poorly recapitulate human tissues and lead to inaccuracies in preclinical data and inefficiencies in drug discovery [136]. This is an important biological limitation because tissues and organs that work together in the body communicate and impact each other’s function. Therefore, in order to sufficiently model the human body for biomedical and pharmaceutical researches, a clear need exists for in vitro models that recapitulate the reproductive system and incorporate its endocrine signaling to the other organs of the body. In this regard, OOAC and human-on-a-chip microfluidic technology devices have recently garnered great attention and offer an alternative platform for animal models to test the efficacy and toxicity of new drugs on several cell types, tissues, and organs within a more biologically relevant environment [21, 142]. This technology is a promising platform to model human diseases and study tissue development in vitro and may significantly affect the future of medicine. Currently, our view of the OOAC and human-on-a-chip is like a jigsaw puzzle with different connecting pieces. However, the pieces will gradually connect and, in the near future, this complicated puzzle will be completed [21, 142].

The reproductive system-on-a-chip (repro-on-a-chip) is promising model to overcome these gaps and provide a new platform that incorporates reproductive sex hormones in preclinical research to address sexual dimorphism in biological processes, disease development, and improve sex-specific drug development [21]. By developing the male and female repro-on-a-chip, an important piece will be added to the human-on-a-chip puzzle, and will raise the hope to provide a suitable in vitro model to study reproductive development, fertility, and reproductive disorders such as implantation disorders or even cancers. In addition, these micro-physiological systems can allow researchers to study the effects of reproductive hormones throughout the body. Moreover, an available dynamic flow microenvironment in the OOAC platform mimics chemophysical signaling between different tissues and provides a controlled microfluidic environment for pharmacokinetic modeling and toxicology studies. This approach is useful for fundamental studies in reproductive biology such as oocyte maturation and modeling pathological conditions of infertility, endometriosis, STD, premature ovarian failure, and polycystic ovary syndrome [143]. Recently, Xiao et al. have integrated female reproductive organs, including the ovaries, fallopian tubes, uterus, and cervix with peripheral organs, including the liver, into a microfluidic system called EVATAR. This EVATAR phenocopies the in vivo female reproductive tract and the hormone profile of the female 28-day menstrual cycle by linking tissues with a sustained circulating flow. Advantages of this technology are the use of human samples (except ovaries that are obtained from rats), long-term culture, and communication between different tissues in a microfluidic platform [21]. However, tissue explants are not expandable and this is a serious challenge for producing scalable chips. The use of female reproductive organoids would allow researchers to fabricate highly scalable and low-cost microfluidic devices for high-throughput assays.

Recent advances in organoid culture systems, including fallopian tubes, endometrium, testicular, and prostate organoids hold promise for the combination of male and female reproductive organoids with other technologies, such as microfluidics technology (Fig. 2). This combined technology would create a multi-organoid-on-a-chip platform and provide human-on-a-chip for clinical applications, drug discoveries, and toxicology studies. In the multi-organoid-on-a-chip systems, the responses of one organoid to drugs or toxins impact the responses of other organoids, which occurs in actual human physiology. These organoids offer insights into the mechanisms of action of drugs or toxins that cannot be predicted by single-organ models. The combination of primary or stem cell-derived human reproductive organoids with other organoids on-a-chip would provide an emerging in vitro platform to study sex-specific differences and the mechanisms of male and female physiology and pathophysiology. This would provide high-value interpretable data to enable the development of more effective and safer drugs, and sex-based therapeutic strategies for precision medicine.

### Challenges and future perspectives

The current version of reproductive organoids faces some challenges; one limitation in the current organoid culture protocols is the requirement of animal-derived and chemically undefined extracellular matrices such as Matrigel. This could limit high-throughput screens and hinder direct transplantation because of medical legislation. Furthermore, tissues display highly anisotropic, heterogeneous, and nonlinear physical properties that depend on their composition, architecture, and pathology [144]. However, isotropic Matrigel is unable to recapitulate the in vivo dynamic changes. To address these issues, hybrid polyethylene glycol (PEG) hydrogels that can change their biophysical and biochemical properties on demand may serve as next-generation designer matrices for organoid production [145]. The lack of stroma, blood vessels, innervation, and immune cells could be a hindrance to organoid research. Development of intestinal organoids with a functional enteric nervous system [146], human pancreatic cancer organoids with matched stromal and immune cells [147], and human prostate organoid co-cultured with prostate stromal cells [148] are good examples of the development of more complex organoid-based structures. Tackling these challenges will open new avenues for biomedical research. Other innovations, such as the use of a bioreactor and optimizing media composition, may minimize variation in culture conditions. Providing a scaffold of biomaterials along with combining microfluidics and organoid technologies may improve tissue architecture. Recent advances in CRISPR/Cas9 and gene editing technologies [149], and single-cell RNA-seq technologies [150] as well as 3D live-cell imaging using light sheet microscopy [151] will greatly accelerate applicability of organoids in biomedical research.

## Conclusion

Despite current limitations, organoids provide an exciting era as in vitro models of the reproductive system that allow for reproductive biology research, accurate modeling of reproductive organs, clinical decision-making, and individualized medicine for patients with gynecologic cancers and diseases such as endometriosis. In addition, bio-banked organoids can be used for drug screening or in vitro trials to predict individual patient drug responses. Advances on cutting-edge technologies will doubtless improve this novel tool for clinical application.

## Data Availability

All data supporting the conclusion of this article are included in this published article.

## Abbreviations

STD: Sexually transmitted disease
ECM: Extracellular matrix
PID: Pelvic inflammatory disease
ALI: Air-liquid interphase
OEC: Oviductal epithelial cells
EGF: Epidermal growth factor
FGF: Fibroblast growth factor
FTE: Fallopian tube epithelium
CGH: Comparative genomic hybridization
CTB: Cytotrophoblast
EVT: Extravillous
STB: Syncytiotrophoblast
HLA: Human leukocyte antigen
C19MC: Chromosome 19 miRNA cluster
BMP4: Bone morphogenetic protein 4
HESC: Human embryonic stem cell
*VCTB*: *Villous CTB*
PC: Principal component
LC-MS/MS: Liquid chromatography-tandem mass spectrometry
PSG: Pregnancy-specific glycoprotein
EPIL: Early placental insulin-like
HCG: Human chorionic gonadotropin
GDF15: Growth differentiation factor 15
KISS1: Kisspeptin
CSH1: Chorionic somatomammotropin hormone 1
EVTM: EVT differentiation medium
CF: Cystic fibrosis
CFTR: CF transmembrane conductance regulator
MTOR: Mammalian target of rapamycin
RTI: Reproductive tract infection
HIV: Human immunodeficiency virus
IUD: Intrauterine device
UEC: Uterine epithelial cell
RWV: Rotating wall vessel
OOAC: Organ-on-a-chip
ZIKV: Zika virus
EOC: Epithelial ovarian cancer
HGSC: High-grade serous carcinoma
EMT: Epithelial-to-mesenchymal transition
TCGA: The cancer genome atlas
WGS: Whole genome sequencing
WES: Whole exome sequencing
PDO: Patient-derived organoid
IHC: Immunohistochemistry
VP: Verteporfin
PDT: Photodynamic therapy
MSI: Microsatellite instability
SCNA: Somatic copy number alteration
cCCC: Cervical clear cell carcinoma
AS: Asherman’s syndrome
IUA: Intrauterine adhesions
RIF: Repeated implantation failure
EA: Endometrial atrophy
ADR: Adverse drug reactions
CYP: Cytochrome P450
GH: Growth hormone
GHR: GH receptor
PEG: Polyethylene glycol
